# Introducing differentiated service delivery models for tuberculosis treatment: a pilot project to inform national policy in Uganda

**DOI:** 10.1002/jia2.26483

**Published:** 2025-07-07

**Authors:** Odile Ferroussier‐Davis, Deus Lukoye, Susan Alwedo, Mary N. Mudiope, Joanitah Nalunjogi, James Bruce Kirenga, Joseph N. Kabanda, Julius N. Kalamya, Benson Nasasira, Estella Birabwa, Seyoum Dejene, Miriam Murungi, Immaculate Ddumba, Brittany Moore, Aldomoro Burua, Henry Luzze, Ebony Quinto, Moorine Sekadde, Raymond Byaruhanga, Patrick Ajuna, Ivan Arinaitwe, Cordelia Katureebe, Proscovia Namuwenge, Michelle R. Adler, Stavia Turyahabwe

**Affiliations:** ^1^ U.S. Centers for Disease Control and Prevention Global Health Center, Division of Global HIV & TB Atlanta Georgia USA; ^2^ U.S. Centers for Disease Control and Prevention Global Health Center, Division of Global HIV & TB Kampala Uganda; ^3^ The AIDS Support Organization (TASO) Kampala Uganda; ^4^ Infectious Diseases Institute (IDI) Kampala Uganda; ^5^ Makerere University Lung Institute (MLI) Kampala Uganda; ^6^ United States Department of Defense (DOD) Kampala Uganda; ^7^ United States Agency for International Development Kampala Uganda; ^8^ Ministry of Health, National TB and Leprosy Program Kampala Uganda; ^9^ Ministry of Health, AIDS Control Program Kampala Uganda; ^10^ U.S. Centers for Disease Control and Prevention Global Health Center, Division of Global HIV & TB Mbabane Eswatini

**Keywords:** community health services, delivery of healthcare, differentiated care, retention in care, tuberculosis, Uganda

## Abstract

**Introduction:**

Differentiated service delivery (DSD) models aim to tailor health services delivery to clients’ preferences and clinical characteristics while reducing the burden on health systems. In Uganda, DSD models developed for HIV care were adapted to the tuberculosis (TB) services context to mitigate disruptions from the COVID‐19 pandemic and inform national efforts to improve TB care.

**Methods:**

Beginning in April 2021, four facility‐based and five community‐based DSD models were implemented in 28 TB clinics in Kampala and Soroti Regions. All clients in the intensive (months 1–2) and continuation (months 3–6) phases of treatment were eligible. Client preference and clinician concurrence determined model choice. All models allowed TB medication dispensing intervals ranging from biweekly to multi‐month dispensing (MMD; ≥ 2 months). Data abstracted in December 2022 from TB registers and DSD enrolment tracking tools at 21 of 28 implementing facilities were used to evaluate the intervention. The TB treatment success rate (i.e. proportion cured or who completed treatment, vs. those who died, failed, were lost‐to‐follow‐up or had no recorded outcome) in the DSD cohort was compared to facilities’ 2018–2019 results using Fischer's exact test.

**Results:**

Most facilities offered one (Kampala) or two (Soroti) facility‐based models and one community‐based model. Among 1864 TB clients enrolled between April 2021 and March 2022, 1822 (97.7%) used ≥ 1 DSD models; 210/1822 (11.5%) ever switched models. Overall, 70.5% (1284/1822) of clients enrolled in ≥ 1 facility‐based model and 40.5% (737/1822) in ≥ 1 community‐based model. The use of community‐based models increased during the continuation phase. Facility‐Based Individual Management and Home Delivery were the most‐used models. In the intensive phase, the longest medication dispensation interval was biweekly for 50.0% of patients, monthly for 41.3% and MMD for 8.8%. During the continuation phase, the longest interval was biweekly for 0.6%, monthly for 71.7% and MMD for 27.6%. Overall, 1582/1864 (84.9%) clients were successfully treated, compared to 858/1177 (72.9%) in 2018–2019 (*p* < 0.001). Seven (0.4%) patients failed treatment, 32 (1.7%) were lost to follow‐up, 101 (5.4%) died and 142 (7.6%) were not evaluated.

**Conclusions:**

TB DSD models were successfully implemented. TB treatment outcomes under DSD compared favourably to historical outcomes. Investigating factors affecting MMD use and model choice could further inform programme design.

## 1 INTRODUCTION

Differentiated service delivery (DSD) refers to person‐centred outpatient clinical care that reflects the preferences and expectations of various groups of clients while reducing unnecessary burdens on health systems [1]. DSD models can consider the clinical (e.g. virally suppressed or not if living with HIV) or behavioural (e.g. adherent to treatment or not) characteristics of the patient, specific populations (e.g. adults, children) and context (e.g. urban, rural). They tailor service delivery along several dimensions: the services offered, timing or frequency of service delivery, delivery location and staff cadre offering the service [2]. This allows for the creation of delivery approaches that improve access to and uptake of services, reduce patient costs [3] and enhance the overall quality of care for targeted populations.

Since 2017, DSD models have become an integral part of HIV care programmes in many countries, including Uganda [4, 5, 6, 7, 8], evolving continually to meet the changing needs of people living with HIV (PLHIV). Evidence exists that DSD models have optimised HIV prevention and treatment services, including antiretroviral therapy (ART), while also streamlining care in contexts of limited human resources and infrastructure [5]. In addition, DSD models appear to have improved treatment outcomes for PLHIV, particularly retention in care and viral suppression [9, 10]. In contrast, DSD use in tuberculosis (TB) care has been limited primarily to the provision of TB preventive treatment among PLHIV [2, 11, 12, 13, 14].

In Uganda, the TB DSD pilot project was developed to mitigate disruptions to healthcare service delivery by the COVID‐19 pandemic, as containment measures, including movement restrictions and staff reassignments, affected the provision of care. In line with international guidelines, standard care for drug‐susceptible TB patients in Uganda [15] requires frequent health facility visits. Treatment includes a 2‐month intensive phase of isoniazid, rifampicin, pyrazinamide and ethambutol, followed by a 4‐month continuation phase of isoniazid and rifampicin. During month 1, patients collect medications in 2‐week increments. In months 2–6, drug dispensing occurs monthly. Patients collect their medications from the facility's pharmacy after being seen by the TB nurse for clinical monitoring. At months 2, 5 and 6, the visit to the facility also includes the collection of a specimen for sputum smear testing, a negative month‐6 smear serving to confirm cure. Directly observed therapy (DOT) is provided in facility by a health worker (primarily to hospitalised patients) or in community by a trained community worker or a family member for treatment adherence.

At the pandemic outset, there were concerns that the negative impacts of COVID‐19 on global TB programmes would reverse gains made over the past decade to reduce TB morbidity and mortality [16]. Ensuring the continuity of TB services by adopting innovative approaches for screening, diagnosis, prevention and treatment [17] was critical. In collaboration with the Uganda Ministry of Health (MOH), notably the National TB and Leprosy Program (NTLP), and with funding from the U.S. Centers for Disease Control and Prevention (CDC), the Infectious Diseases Institute (IDI) (Kampala) and The AIDS Support Organization (TASO) (Soroti) implemented a pilot project aimed at differentiating TB service delivery, innovated around the approaches successfully used for HIV care. For TB treatment, the project focused on lessening the burden of biweekly or monthly facility‐based medication dispensation. Lessons from this pilot were to inform MOH efforts to develop national guidelines on TB DSD models.

## 2 METHODS

### 2.1 Implementation approach

The project's long‐term goal was to test innovative strategies to improve the quality of TB services using a patient‐centred approach. Its short‐term purpose was to ensure the continuity of TB services during the COVID‐19 pandemic. The project was implemented in 28 health facilities managing rifampicin‐sensitive TB patients across 16 districts in two localities, Kampala and Soroti, supported by partners implementing CDC‐funded comprehensive HIV/TB projects. Selected sites were high‐volume facilities not implementing any form of TB services differentiation; in Kampala, the project also targeted sites that performed sub‐optimally on > 1 NTLP indicators in 2019 (Table S1) to learn lessons applicable to settings where patients stand to gain most from improvements in service delivery.

Project rollout occurred in March−November 2021 in Kampala and in April−August 2021 in Soroti. TB DSD models proposed for implementation matched the facility‐ and community‐based models developed for HIV care in Uganda [5] (Table 1), and sites’ initial offering largely reflected the models already operating for PLHIVs. Facility‐Based Individual Management (FBIM) was the model closest to standard TB care. All TB patients managed at each site were eligible for enrolment into DSD, regardless of age, HIV status, clinical condition or phase of TB treatment, with model options contingent on whether a patient was categorised as stable or unstable. “Unstable” patients included those in the intensive phase, lacking reliable contact information, who had missed ≥ 2 weeks of treatment or virally unsuppressed (> 1000 copies/ml) if living with HIV. All models allowed medication dispensation intervals ranging from biweekly to multi‐month dispensing (MMD; 2 months in Kampala, 2–4 months in Soroti). Suitable DSD models for a patient were proposed by clinicians based on the patient's clinical characteristics. When multiple models were suitable, enrolment considered patient preference. Model switches, at patient's request or upon clinician's recommendation, were possible during and between treatment phases. To help staff track DSD enrolment, project‐specific electronic and paper‐based tools were introduced for data elements not available in the routine health information system.

**Table 1:** Description of differentiated service delivery (DSD) models for tuberculosis (TB) treatment considered for implementation and eligibility in 21 health facilities, Kampala and Soroti regions, Uganda, 2021–2022

Model	Description	Eligible clients	When	Where	Who	What
FACILITY						
Facility‐Based Individual Management (FBIM)	A more intensive model for patients needing in‐person review at their drug pick‐ups	S, U	Intensive and continuation phase	TB clinic and pharmacy in health facility	TB nurse Pharmacist Cough monitor Community‐based volunteer	Clinical review TB drug refills Adherence support Psychosocial support Laboratory tests
Fast‐Track Drug Refill (FTDR)	Client picks up TB drugs from pharmacy on scheduled appointment date without first seeing clinician	S	Continuation phase	Pharmacy in health facility	Pharmacist/dispenser	TB drug refills without seeing the clinician alternating with scheduled clinician reviews every 1−2 months
Refill Alignment with Comorbidities (RAC)	Client receives TB drugs along with medication for another chronic condition (e.g. HIV); appointment dates are harmonised	S	Continuation phase	Pharmacy in health facility	Pharmacist/dispenser	TB drug refills
Facility‐Based Group (FBG)	Patient attending specialty clinics (e.g. comorbidities, high viral load) is attended to in that clinic	S, U	Intensive and continuation phase	HIV clinic or any other specialty clinic in health facility	Health worker Peers	Adherence support Psychosocial support
COMMUNITY						
Home Delivery (HD)	Cough monitors on motorcycles deliver TB drugs to patients’ home; health workers may ride along to check on unstable patients	S, U	Intensive and continuation phase	Patient home	Motorcycle driver Cough monitor Health worker	TB drug refills Clinical monitoring Adherence support Psychosocial support Sputum collection
Community Drug Distribution Points (CDDP)	TB drugs delivered to location convenient to 5+ patients in given area	S	Continuation phase	Any community location	Health worker Peers Pharmacist	TB drug refills Clinical evaluation by health worker if integrated into HIV outreach programme
Community Pharmacy Refills (CPR)	Patient picks up TB drugs at community retail pharmacy	S	Continuation phase	Retail pharmacy in community	Retail pharmacist	TB drug refills
Community Refill Groups (CRG)	TB drugs resupply distributed through community‐based support groups	S, U		Any community location		TB drug refills
Community Client‐Led Distribution (CCLD)	Delivery of medication at the community level to a community TB group by one of the group members on a rotational basis	S	Continuation phase	Any community location	Peers/expert client	TB drug refills

Abbreviations: S, stable (patient in continuation phase, with valid contact information/address, virally suppressed if living with HIV, with no treatment interruption ≥ 2 weeks); U, unstable (patient in intensive phase, with unreliable contact information, virally unsuppressed if living with HIV, with treatment interruptions ≥ 2 weeks, sputum smear positive at month 2 or month 5).

Importantly, in addition to introducing DSD models for TB treatment, IDI and TASO comprehensively reorganised many aspects of TB diagnosis and care service delivery through a client‐centred lens. For example, rosters of TB patients were assigned to community‐based volunteers supported by mentors to provide ongoing treatment literacy and monitor adherence and side effects. The use of appointment registers and pre‐appointment reminders was strengthened, with same‐day follow‐up for patients failing to appear. Community activities were integrated: for example home deliveries integrated sputum sample collection for treatment follow‐up.

### 2.2 Evaluation

To describe the implementation of DSD models and relate changes in TB service delivery with TB treatment outcomes (defined according to World Health Organization criteria [18] and national guidelines [15]), data were abstracted from TB unit registers and DSD enrolment tracking tools at 21 of the 28 pilot facilities. All TB patients registered in the 21 pilot facilities between the facility's implementation start date (March−November 2021) and March 2022 were included in the implementation cohort, and their deidentified individual‐level data was abstracted retrospectively. As a measure of TB care quality under DSD, we assessed whether bacteriologically confirmed pulmonary (PBC) TB patients received follow‐up sputum smears at months 2, 5 and 6 to monitor progress towards cure, as less frequent encounters or community‐based care might hamper timely sputum collection. To assess the relative performance of DSD compared to traditional service delivery in ensuring treatment success (i.e. cured or completed treatment), a historical cohort of patients registered for TB treatment at the same facilities during the corresponding months of 2018–2019 (i.e. March−November 2018, depending on the facility, until March 2019) was selected. Patients registered in 2020, when the health system was experiencing the greatest pandemic‐related upheavals, were purposively excluded. For the historical comparison cohort, only aggregate (monthly) data were collected from facilities’ TB registers. TB treatment outcomes were abstracted as assigned by facility clinicians.

Data abstraction was performed by research assistants trained on the DSD models, evaluation protocol, field procedures and data sources. The data abstraction censor date was 23 December 2022. Data abstraction tools were piloted at two non‐intervention sites before being finalised and standard operating procedures generated. Abstracted data were entered into an online database with built‐in completeness and accuracy logic checks, hosted on a secure Kobo server.

Models with similar design but different labels across localities (e.g. “home delivery with on‐call support” and “community‐supported refills”) were combined for analysis. Data analysis was performed using Stata software (version 16.1, Stata Corp LLC, College Station, TX, USA). Descriptive statistics including proportions (for categorical variables) and means with standard deviations or medians with interquartile ranges (IQR) (for continuous variables) were computed. Group differences in treatment success rates (between the DSD cohort and the historical cohort as well as for subgroups within the DSD cohort) were tested using chi‐square statistics for categorical variables. All tests were two‐tailed; statistical significance was set at *p* ≤ 0.05.

The evaluation was reviewed and approved by Makerere University School of Public Health's Research and Ethics Committee (SPH‐2022‐254), and the Uganda National Council for Science and Technology (HS3122ES). CDC reviewed the protocol and deemed it not research (CGH‐UGDA‐12/14/21‐3d907). The evaluation was conducted consistent with applicable federal law and CDC policy 45 C.F.R. part 46.102(l)(2), 21 C.F.R. part 56; 42 U.S.C. §241(d); 5 U.S.C. §552a; 44 U.S.C. §3501 et seq.

## 3 RESULTS

### 3.1 Characteristics of patients in implementation cohort

The DSD implementation cohort (Table 2) comprised 1864 TB patients (1026 in Kampala, 838 in Soroti) who initiated treatment at 21 of 28 pilot facilities between the intervention start date (March−November 2021) and 30 March 2022. Fifty‐six percent (1044/1864) were males. Median age was 38 years (IQR: 27–51); 190 (10.2%) patients were ≤ 15 years of age. HIV status was known for 1830 patients (98.2%); 815 (44.5%) were PLHIV. Overall, 1708 (91.6%) were new TB cases; 1781 (95.5%) had pulmonary TB, including 982 (55.1%) with bacteriologic confirmation. Information about DSD enrolment was available for 1822 patients (97.7%). Availability of DSD modality information did not vary significantly by locality, age, sex or HIV status.

**Table 2:** Characteristics of patients receiving treatment for drug‐sensitive tuberculosis (TB) in differentiated service delivery (DSD) implementation cohort in 21 health facilities, Kampala and Soroti regions, Uganda, 2021–2022

Characteristic	Kampala region *N* = 1026 *n* (%)	Soroti region *N* = 838 *n* (%)	Total *N* = 1864 *n* (%)
Sex			
Male	608 (59.3%)	436 (52.0)	1044 (56.0)
Female	418 (40.7)	402 (48.0)	820 (44.0)
Age (years)			
< 15	86 (8.4)	95 (11.4)	181 (9.7)
15−24	119 (11.6)	68 (8.1)	187 (10.0)
25−34	284 (27.7)	112 (13.4)	396 (21.3)
35−44	263 (25.7)	158 (18.9)	421 (22.6)
45−54	156 (15.2)	147 (17.6)	303 (16.3)
55−64	74 (7.2)	98 (11.7)	172 (9.2)
≥ 65	43 (4.2)	158 (18.9)	201 (10.8)
Missing^a^	1	2	3
TB type			
PBC	476 (46.6)	506 (60.4)	982 (52.8)
PCD	511 (50.0)	288 (34.4)	799 (42.9)
EPTB	35 (3.4)	44 (5.2)	79 (4.3)
Missing^a^	4	0	4
Type of TB case			
New	951 (93.5)	757 (91.0)	1708 (92.4)
Relapse	45 (4.4)	60 (7.2)	105 (5.7)
Treatment after failure	3 (0.3)	7 (0.8)	10 (0.5)
Treatment after loss to follow‐up	18 (1.8)	8 (1.0)	26 (1.4)
Missing^a^	9	6	15
HIV status			
Negative	502 (48.9)	513 (61.2)	1015 (54.5)
Known positive	327 (31.9)	258 (30.8)	585 (31.4)
New positive	182 (17.7)	48 (5.7)	230 (12.3)
Unknown	15 (1.5)	19 (2.3)	34 (1.8)

Abbreviations: EPTB, extrapulmonary TB; PBC, pulmonary TB, bacteriologically confirmed; PCD, pulmonary TB, clinically diagnosed.

^a^ Missing values are excluded from percentage calculations.

### 3.2 Model rollout

Table 3 summarises model use across the two localities. Of nine DSD models considered for implementation during the project planning phase, eight were used across the two localities: five (two facility‐based, three community‐based) in Kampala and six (four facility‐based, two community‐based) in Soroti. Most Kampala facilities offered one facility‐based and one community‐based model, while most Soroti facilities offered two facility‐based and one community‐based model.

**Table 3:** Tuberculosis (TB) differentiated service delivery (DSD) model offering and enrolment in 21 health facilities, Kampala and Soroti regions, Uganda, 2021–2022^a^

	KAMPALA		SOROTI		
	Facilities implementing in region	Patients ever enrolled in model in region	Facilities implementing in region	Patients ever enrolled in model in region	Total patients ever enrolled in model
FACILITY					
FBIM	12	799 (77.9%)	9	207 (24.7%)	1006 (54.0%)
FTDR	2	30 (2.9%)	8	248 (29.6%)	278 (14.9%)
RAC	0	0	1	8 (1.0%)	8 (0.4%)
FBG	0	0	2	5 (0.6%)	5 (0.3%)
HD	12	346 (33.7%)	9	352 (42.0%)	698 (37.4%)
COMMUNITY					
CDDP	2	32 (3.1%)	0	0	32 (1.7%)
CPR	1	1 (0.1%)	0	0	1 (< 0.1%)
CRG	0	0	3	24 (2.9%)	24 (1.3%)
CCLD	0	0	0	0	0

Abbreviations: CCLD, community client‐led distribution; CDDP, community drug‐distribution point; CPR, community pharmacy refill; CRG, community refill group; FBG, facility‐based group; FBIM, facility‐based individual management; FTDR, fast‐track drug delivery; HD, home delivery; RAC, refill alignment with comorbidities.

^a^ Patients could enrol in more than one model over the course of TB treatment.

### 3.3 Facility versus community‐based models

Overall, 1284/1822 patients (70.5%) enrolled in at least one facility‐based model and 738/1822 (40.5%) in at least one community‐based model; 1084/1822 (59.5%) patients used only facility‐based models and 538/1822 (29.5%) used only community‐based models. Two hundred patients (11.0%) used at least one facility‐based and one community‐based model over the course of treatment. FBIM and Home Delivery were the facility‐based and community‐based models, respectively, that enrolled the most patients (Table 3).

### 3.4 Model switches

Only 210/1822 patients (11.5%) were recorded as having enrolled in ≥ 2 DSD models over the course of TB treatment. Model switching was more frequent in Kampala (198/1026, 19.3%) than in Soroti (12/838, 1.4%) (*p* < 0.001).

### 3.5 Intensive versus continuation phase

Information about model enrolment disaggregated by TB treatment phase was available for a subset of 1698 patients (93.2%). Enrolment in facility‐based models was stable across treatment phases (approximately 70.0%); use of community‐based models increased from 33.5% (569/1698) during the intensive phase to 44.0% (668/1519) during the continuation phase (*p* < 0.001, data not reflected in tables).

### 3.6 Medication dispensation

Information about drug dispensation schedules was available for 1838 patients (98.6%) during the intensive phase and 1671 patients (89.6%) during the continuation phase. In the intensive phase, a patient's longest dispensation interval was biweekly for 50.0% of patients, monthly for 41.3% and MMD for 8.8%. In the continuation phase, the longest interval was biweekly for 0.6%, monthly for 71.7% and MMD for 27.6%. Overall, 483/1864 patients (26.3%) ever received MMD during TB treatment. The likelihood of ever receiving MMD was comparable whether patients enrolled only in facility‐based models (26.8%), only in community‐based models (25.7%) or in both model types (24.5%).

Non‐digital community DOT was the most commonly used treatment observation method among the DSD cohort (1620, 86.9%). Only 35 patients received facility‐based DOT. Thirty‐nine patients, 36 from one Kampala facility, received digital community DOT.

### 3.7 Follow‐up smears

Among patients recorded as having PBC TB, 536 (54.6%) had a month‐6 sputum smear documented in the TB register; 432/982 (44.0%) had follow‐up smears at all three time points. The proportions for month 6 and all three time points were 59.2% (282/476) and 55.2% (263/476) in Kampala and 50.2% (254/506) and 33.4% (169/506) in Soroti, respectively. Among PBC TB patients enrolled in at least one community‐based DSD model, 55.9% (214/383) had a month‐6 smear, compared to 52.9% (376/669) among those enrolled in at least one facility‐based model (*p* = 0.92). Finally, 66.4% of PBC TB patients on MMD during the continuation phase had a month‐6 smear, compared to 56.5% of patients who got more frequent resupplies (*p* < 0.01).

### 3.8 TB treatment outcomes

Overall, 1582/1864 patients (84.9%) in the DSD cohort were successfully treated (cured or completed treatment), compared to 858/1177 patients (72.9%, *p* < 0.01) at the same facilities in 2018–2019 (Tables 4 and S2). The proportion of TB patients with undocumented outcome/not evaluated was lower in the DSD cohort than in the historical cohort. In the DSD cohort, the probability of treatment success was higher in Kampala than in Soroti, and in women compared to men, but differences in treatment success rates by HIV status and TB patient type were not statistically significant (Table 5).

**Table 4:** Tuberculosis (TB) treatment outcomes for individual models and for overall differentiated service delivery (DSD) and comparison cohorts in 21 health facilities, Kampala and Soroti regions, Uganda, 2021–2022^a^

	Facility‐based models				Community‐based models				Overall DSD cohort	Historical comparison cohort
	FBIM	FTDR	RAC	FBG	HD	CDDP	CPR	CRG		
TB treatment outcome	*N* = 1006^b^ *n* (%)	*N* = 278^b^ *n* (%)	*N* = 8^b^ *n* (%)	*N* = 5^b^ *n* (%)	*N* = 698^b^ *n* (%)	*N* = 32^b^ *n* (%)	*N* = 1^b^ *n* (%)	*N* = 24^b^ *n* (%)	*N* = 1864 *n* (%)	*N* = 1177 *n* (%)
Cured/completed	843 (83.8)	242 (87.1)	5 (62.5)	5 (100)	629 (90.1)	31 (96.9)	1 (100)	21 (87.5)	1582 (84.9)^c^	858 (72.9)^c^
Lost to follow‐up	15 (1.5)	7 (2.5)	0	0	7 (1.0)	0	0	3 (12.5)	32 (1.7)	100 (8.5)
Failed	4 (0.4)	2 (0.7)	0	0	1 (0.1)	0	0	0	7 (0.4)	9 (0.8)
Died	70 (7.0)	10 (3.6)	1 (12.5)	0	21 (3.0)	1 (3.1)	0	0	101 (5.4)	82 (7.0)
No recorded outcome^d^	74 (7.4)	17 (6.1)	2 (25.0)	0	40 (5.7)	0	0	0	142 (7.6)	128 (10.9)

Abbreviations: CDDP, community drug‐distribution point; CPR, community pharmacy refill; CRG, community refill group; FBG, facility‐based group; FBIM, facility‐based individual management; FTDR, fast‐track drug delivery; HD, home delivery; RAC, refill alignment with comorbidities.

^a^ Patients in DSD cohort could enrol in more than one DSD model over the course of TB treatment.

^b^ DSD cohort patients ever enrolled in the model during either intensive or continuation phase of TB treatment.

^c^ The *p*‐value for the chi‐square test of significance comparing the treatment success rates (proportion of patients recorded as cured or having completed treatment) was < 0.0001.

^d^ Outcome field in TB register left blank or patient marked as not evaluated.

**Table 5:** Tuberculosis (TB) treatment outcomes in the differentiated service delivery (DSD) cohort in 21 health facilities, Kampala and Soroti regions, Uganda, 2021–2022, by region, sex, HIV status and type of TB case

	Unsuccessful outcome	Successful outcome	Relative risk of experiencing a successful outcome			
	*n* (%)	*n* (%)	Risk ratio	95% CI	*X* ^2^	*p* value
**By region**						
Soroti (*n* = 838)	136 (16.6)	699 (83.4)	Reference			
*LTFU*	22 (2.6)					
*Failed*	4 (0.5)					
*Died*	53 (6.3)					
*Not recorded* ^a^	60 (7.2)					
Kampala (*n* = 1026)	126 (14.0)	883 (86.0)	1.04	1.00−1.09	5.41	0.0200
*LTFU*	10 (1.0)					
*Failed*	3 (0.3)					
*Died*	48 (4.7)					
*Not recorded* ^a^	82 (8.0)					
**By sex**						
Male (*n* = 1044)	178 (17.0)	866 (83.0)	Reference			
*LTFU*	15 (1.4)					
*Failed*	5 (0.5)					
*Died*	65 (6.2)					
*Not recorded* ^a^	93 (8.9)					
Female (*n* = 820)	104 (12.7)	716 (87.3)	1.05	1.01−1.09	6.82	0.0090
*LTFU*	17 (2.1)					
*Failed*	2 (0.2)					
*Died*	36(4.4)					
*Not recorded* ^a^	49 (6.0)					
**By HIV status**						
HIV negative (*n* = 1015)	138 (13.6)	877 (86.4)	Reference			
*LTFU*	19 (1.9)					
*Failed*	4 (0.4)					
*Died*	47 (4.6)					
*Not recorded* ^a^	68 (6.7)					
HIV positive (*n* = 815)	130 (16.0)	685 (84.0)	0.97	0.94−1.01	2.01	0.1567
*LTFU*	11 (1.3)					
*Failed*	2 (0.2)					
*Died*	51 (6.3)					
*Not recorded* ^a^	66 (8.1)					
**By TB patient type**						
Retreatment (*n* = 141)	29 (10.6)	112 (79.4)	Reference			
*LTFU*	3 (2.1)					
*Failed*	2 (1.4)					
*Died*	11 (7.8)					
*Not recorded* ^a^	13 (9.2)					
New (*n* = 1708)	252 (14.8)	1456 (85.2)	1.07	0.98−1.17	3.42	0.0646
*LTFU*	29 (1.7)					
*Failed*	5 (0.3)					
*Died*	90 (5.3)					
*Not recorded* ^a^	128 (7.5)					

Abbreviations: CI, confidence interval; LTFU, lost to follow‐up.

^a^ Outcome field in TB register left blank or patient marked as “not evaluated.”

## 4 DISCUSSION

This evaluation documents the successful piloting of TB DSD models in Uganda in the context of the COVID‐19 epidemic. The national‐level TB treatment success rate increased from 78.3% in 2018–2019 to 88.9% in 2021 [19]. Results at pilot sites show a similar trend, even with flexibilisation of medication pick‐up schedules and location, including in Kampala sites selected for their suboptimal pre‐intervention performance on a range of TB indicators. Improvements in the treatment success rate appear to be due primarily to improved tracking of patients throughout treatment, as measured in the decreased proportion of patients lost‐to‐follow‐up, not evaluated or with no recorded outcome in the DSD cohort. Examples of interventions that improved retention and tracking included strengthening the role of community‐based volunteers, conducting cohort reviews and developing individual follow‐up plans for each client. These methods were integrated into routine practice at pilot sites and expanded to other facilities. Their implementation beyond the evaluation phase demonstrates their feasibility. However, resource availability remains a critical factor for long‐term adoption.

The Uganda approach to DSD for TB care is inclusive: it does not limit eligibility to patients deemed “stable” (clinically or socially) and modulates the intensity of follow‐up to match patients’ needs and preferences. FBIM, the model that is closest to standard‐of‐care for TB treatment and was the most‐used facility‐based model during the pilot, still allows flexibility through MMD to decrease the frequency of clinic attendance. In addition, FBIM varies from standard of care, because participation in FBIM is chosen by, rather than imposed upon, TB patients. Home Delivery, the most‐used community‐based model, is particularly well‐suited for sicker patients who struggle to attend health facilities for drug pick‐ups and clinical monitoring. Uganda's approach frames DSD as a person‐centred approach tailored to each person's needs, not as a reward for those showing good adherence or clinical improvement.

The pilot facilities provide TB services to drug‐susceptible TB patients only. Therefore, the evaluation could not ascertain how DSD could serve patients with drug‐resistant TB, who, facing even greater adherence challenges, stand to benefit considerably from differentiated care. All‐oral short‐course regimens for drug‐resistant TB, now available in Uganda, create opportunities for differentiation mirroring those offered to patients with drug‐susceptible TB. However, resource‐related factors (e.g. drug availability) need to be considered.

While the project intended to offer the same broad range of DSD options available for HIV care, evaluation data show that TB patients’ DSD enrolment was highly concentrated in a few models. Counterintuitively, more community‐based models were used in Kampala (mostly urban/peri‐urban, with working/mobile population) and more facility‐based models in Soroti (more rural, less densely populated). It is unclear whether this reflected patients’ preferences or the difficulty of delivering community care in rural settings. Evidence of the latter may be seen in the plurality of Soroti patients opting for community‐based models, while patients in Kampala more frequently opted for facility‐based models. This suggests that the geographic context of service delivery might affect both patient preferences and health facilities’ ability to offer certain DSD options. In addition, the pilot showed that community‐based group models previously shown to work well for patients on ART [4, 6, 7] did not translate easily to the context of TB service provision. Because there are far fewer TB patients in a given locality at a given time and TB treatment is relatively short, Community Drug Distribution Points were more difficult to set up for TB than for ART. Similarly, the Community Pharmacy Refills network for antiretrovirals distribution, which uses local retail pharmacies as medication pick‐up points, struggled to onboard new pharmacies in areas where HIV‐negative TB patients reside. In addition, site staff reported during project monitoring visits that some clients expressed fears of stigma and loss of privacy in association with community models. This is a reminder for planners and policymakers that a broad range of factors impact programme design and that “one coat will not fit all” across diseases. Investigating these factors may be informative prior to expanding DSD to other settings in Uganda and beyond to strike the right balances between patient needs and preferences and feasibility. Some barriers might disappear over time, as networks of community distribution points, be they retail pharmacies or patient support groups, mature and become established beyond the shorter‐term perspective of this pilot intervention. Greater patient enrolment in a broader range of models will in turn generate more granular data to evaluate the relative performance of individual models.

The evaluation found that few patients switched models over time. This could indicate that the models offered by the facilities, coupled with the MMD option, met patients’ needs and preferences. It could also reflect the fact that individual sites offered a limited range of model options, although all sites offered at least two (one facility‐based, one community‐based). Yet, another explanation might be the relatively short span of TB treatment (6 months), compared to lifelong ART, which affords less time (and less need) to plan for transitions to different models [20]. TB patients’ relative stability may assuage concerns about the need for substantial health staff effort to track patients’ changes of modality over time.

Overall, approximately a quarter of patients in the DSD cohort benefited from MMD at some point during TB treatment. This relatively low estimate may reflect health workers' lack of awareness or initial reluctance to vary from standard practice. It could also correlate with site‐level tensions on TB drug stocks that may have constrained dispensing to ensure that all patients were served. In this evaluation, we were unable to disaggregate the analysis by length of MMD interval (2 months vs. longer). As evidence becomes available about longer dispensing intervals (e.g. 4 months during the continuation phase), especially among patients on ART, combined with the introduction of shorter treatment regimens, there may be opportunities to further decrease the burden of treatment on TB patients.

Across all DSD models, evaluation findings highlight the need to reinforce to TB patients and to health staff the importance of performing follow‐up smear evaluations at intervals set by national TB guidelines and recording their results, so the paramount goal of successful treatment is not jeopardised. Strategies could involve strengthening community sputum sample collection, aligning sputum collection with clinic visits, and issuing reminders to both staff and patients. Importantly for DSD, patients in less intensive modalities, particularly those receiving MMD or enrolled in community models, were no less likely to undergo follow‐up sputum smear testing than patients receiving facility‐based care or more frequent drug distribution.

This evaluation had several limitations. First, the design did not include a set of control sites. Therefore, we cannot dismiss the possibility that, although statistically significant, improved treatment success rates in the DSD cohort reflected a national or locality‐wide improvement in TB outcomes attributable to other MoH‐ or donor‐backed efforts. Second, it is difficult to isolate the effect of DSD from that of other concurrent modifications to TB case management (e.g. the use of appointment registers and pre‐appointment reminders), as both TASO and IDI comprehensively reorganised their approaches through a patient‐centred lens. Some of these interventions, including the provision of intensive education and psychosocial support to patients, unmeasured in our evaluation, may well be essential to the success of DSD. Third, included sites represent a variety of care settings, but their small number might limit generalisability. Additionally, the use of aggregate data for the historical cohort hampers our ability to assess whether the two cohorts’ composition (e.g. age, HIV status) was similar. Moreover, as in any evaluation relying on retrospectively abstracted programmatic data, we could not ascertain whether the absence of information (e.g. about follow‐up smears) reflected the non‐provision of a service or failure to record it appropriately. Similarly, the “true” status of patients with no TB treatment outcome or categorised as lost‐to‐follow‐up was not ascertained. Furthermore, without detailed information about individual patients’ clinical status, we could not control for disease severity when assessing the relationship between model participation and treatment outcomes. Finally, the scope of the evaluation did not include an assessment of the DSD approach's sustainability or scale‐up cost implications, nor of its impact on participating sites’ patient management practices beyond the intervention phase.

## 5 CONCLUSIONS

Uganda's pilot of DSD for TB treatment to mitigate the impact of the COVID‐19 pandemic while improving the quality of TB care built on experience gained in HIV care. Treatment outcomes under DSD models compare favourably with pre‐pandemic results in the same facilities. Although not without limitations, this evaluation adds to the evidence base about the differentiation of services by national TB programmes to improve quality, efficiency, impact and potentially patient satisfaction. As models mature and more data become available to evaluate effectiveness, investigating patient and staff perspectives, and costs, efficiency and sustainability, will also be needed.

## COMPETING INTERESTS

The authors have no competing interests to declare.

## AUTHORS’ CONTRIBUTIONS

*Funding acquisition*: BM, DL, JK and JNK. *Principal investigators*: MNM and ST. *Conceptualization of project*: AB, BM, CK, DL, EB, EQ, HL, IA, ID, JK, JNK, MM, MS, PA, PN, RB, SA, SD and ST. *Project methodology/protocol development*: AB, BM, CK, DL, EB, EQ, HL, IA, ID, JK, JNK, MM, MS, OF‐D, PA, PN, RB, SA, SD and ST. *Data collection/abstraction*: JBK, JN, MNM and SA. *Data analysis*: BN, JBK, JN and OF‐D. *Manuscript drafting and finalisation*: OF‐D. *Manuscript review and editing*: BM, BN, DL, JBK, JK, JNK, MM and MNA. *Project administration*: BN, IA, PA, PN and ST. *Project supervision*: AB, CK, DL, EQ, HL, IA, JK, JNK, MNM, MS, PA, PN, RB, SA and ST. All authors read and approved the final manuscript.

## FUNDING

This project was supported by the CARES Act through the U.S. Centers for Disease Control and Prevention (CDC). It was also supported by the President's Emergency Plan for AIDS Relief (PEPFAR) through the U.S. Centers for Disease Control and Prevention (CDC) under the terms of Cooperative Agreements GH002022 and GH002066.

## DISCLAIMER

The findings and conclusions are those of the authors and do not necessarily represent the official position of the funding agencies.

## Supporting information




**Table S1**: Selection criteria for health care facilities included in the differentiated service delivery (DSD) pilot project in Kampala and Soroti regions, Uganda, 2021‐2022.
**Table S2**: Tuberculosis (TB) treatment outcomes by category of differentiated service delivery (DSD) model in 21 health facilities, Kampala and Soroti regions, Uganda, 2021‐2022.

## Data Availability

The data that support the findings of this study are available on request from the corresponding author. The data are not publicly available due to privacy or ethical restrictions.
