# Supplementary material for: Introducing differentiated service delivery models for tuberculosis treatment: a pilot project to inform national policy in Uganda
Source: J Int AIDS Soc. 2025 Jul 7;28(Suppl 3):e26483. doi: 10.1002/jia2.26483 (PMC12232480; doi:10.1002/jia2.26483)
Supplement: Supplementary file 1 — Table S1: Selection criteria for health care facilities included in the differentiated service delivery (DSD) pilot project in Kampala and Soroti regions, Uganda, 2021‐2022. Table S2: Tuberculosis (TB) treatment outcomes by category of differentiated service delivery (DSD) model in 21 health facilities, Kampala and Soroti regions, Uganda, 2021‐2022. [file JIA2-28-e26483-s001.docx]

**Table S1. Selection criteria for health care facilities included in the differentiated service delivery (DSD) pilot project in Kampala and Soroti regions, Uganda, 2021-2022**

|  | **Kampala** | **Soroti** |
| --- | --- | --- |
| **High volume facilities** | - Facilities cumulatively account for >40% of tuberculosis (TB) cases notified in the district in 2019 | - Facilities cumulatively account for >40% of TB cases notified in the district in 2019 |
| **Subpar performance on >1 national TB indicators** | - Reached <80% of 2019 annual TB detection target OR - In the 2018 annual TB cohort - TB treatment success rate^†^ <85% OR - >5% died OR - >5% were lost to follow-up OR - Among child contacts of registered TB cases: - <50% contacts identified were reached OR - <70% contacts identified were screened OR - <70% contacts eligible for TB preventive treatment (TPT) were initiated |  |

**†** *Treatment success includes patients whose TB treatment outcomes are recorded as “cured” or “completed treatment”.*

**Table S2. Tuberculosis (TB) treatment outcomes by category of differentiated service delivery (DSD) model in 21 health facilities, Kampala and Soroti regions, Uganda, 2021-2022**

|  | **Patients enrolled in…** | | | | |
| --- | --- | --- | --- | --- | --- |
| **Treatment outcome (n, %)** | **≥1 facility-based**  **model**  (n=1,284) | **≥1 community-based model**  (n=738) | **Only facility-based models**  (n=1,084) | **Only community-based models**  (n=538) | **Both facility- and community-based models**  (n=200) |
| Cured/completed | 1,083 (84.3) | 665 (90.1) | 895 (82.6) | 477 (88.7) | 188 (94.0) |
| Lost to follow-up | 21 (1.6) | 10 (1.4) | 21 (1.9) | 10 (1.9) | 0 |
| Failed | 6 (0.5) | 1 (0.1) | 6 (0.6) | 1 (0.2) | 0 |
| Died | 81 (6.3) | 22 (3.0) | 75 (6.9) | 16 (3.0) | 6 (3.0) |
| No recorded outcome^¥^ | 93 (7.2) | 40 (5.4) | 87 (8.0) | 34 (6.3) | 6 (3.0) |

^¥^ *Outcome field in TB register left blank or patient marked as Not evaluated*
